# Prevalence of malaria, anemia and associated factors among school children in Hawassa city, Sidama, Ethiopia

**DOI:** 10.1371/journal.pone.0327378

**Published:** 2025-07-17

**Authors:** Alia Gena, Solomon Asnake, Tadesse Menjetta

**Affiliations:** 1 College of Medicine and Health Sciences, Department of Medical Laboratory Science, Arba Minch University, Arba Minch, Ethiopia; 2 College of Medicine and Health Sciences, School of Medical Laboratory Science, Hawassa University, Hawassa, Ethiopia; PLOS: Public Library of Science, ETHIOPIA

## Abstract

**Background:**

The recurrent occurrence of malaria and anemia are leading causes of morbidity and mortality in children especially in sub-Saharan Africa. In tropical regions, malaria is a major contributor to anemia which occurs due to reduced hemoglobin levels caused by hemolysis of infected and uninfected red blood cells and as a result bone marrow dyserythropoiesis. Though malaria and anemia are two interlinked health problems among school children, there is scarce information about the issue in the study area. Hence the current study aimed to assess the prevalence of malaria and anemia and associated factors among school children in Hawassa City, Sidama, Ethiopia.

**Method:**

A cross-sectional study was conducted from April to June 2024 in selected public primary schools in Hawassa City, recruiting 329 children. Socio-demographic data were collected using a pretested questionnaire. Thick and thin blood films were prepared for microscopic examination of malaria parasites, and parasite counts were conducted. A rapid diagnostic test was also performed for malaria diagnosis. A digital hemoglobinometer was used to determine hemoglobin levels and assess anemia prevalence. Data were analyzed using SPSS version 27, with bivariate and multivariate logistic regression performed. The strength of association was determined by computing adjusted odds ratios with 95% confidence intervals, and a p-value of <0.05 was considered statistically significant.

**Result:**

Malaria and anemia were present in 8.5% and 9.4% of the children, respectively while, 29% of anemic children were also infected by malaria. The odds of having anemia were highest in children with malaria (AOR = 4.983, 95% CI: 1.067–23.265), previous history of malaria (AOR = 9.121, 95% CI: 1.686–49.336). Using insecticide treated-net (AOR = 0.024, 95% CI: 0.001–0.755), knowledge of malaria transmission (AOR = 0.205, 95% CI: 0.049–0.854),has significantly reduced malaria risk, highlighting the role of preventive practices and awareness. Meal frequency (AOR = 6.243, 95% CI: 1.956–19.923), malaria infection (AOR = 13.258, 95% CI: 3.188–55.139), and history of wasting (AOR = 5.760, 95% CI: 2.059–16.112) were identified as significant risk factors of anemia.

**Conclusion:**

This study found that 8.5% of school children in Hawassa City had malaria and 9.4% were anemic, indicating a mild public health concern. A strong association was observed between the two conditions: malaria-infected children were over 13 times more likely to be anemic. These findings highlight the need for integrated malaria prevention and nutrition programs. Interventions should focus on ITN use, improving dietary practices, and identifying asymptomatic malaria carriers to reduce the burden of both diseases.

## Introduction

Malaria is life threatening disease principally found in tropical countries. It is a vector-borne disease that is transmitted by different species of *Anopheles* mosquitoes. Five species of *Plasmodium* can cause the disease in humans. Among the five Plasmodium species, *P. falciparum, P. vivax, P. malariae*, and *P. ovale* are the primary parasites that infect humans, with *P. falciparum* and *P. vivax* posing the greatest threat. *P. knowlesi*, which naturally infects macaques, has recently been recognized as a zoonotic species that can also cause malaria in humans. [1]. Due to several factors such as the type of local mosquitoes, seasonal conditions, migration, and immune status, the risk of infection varies from place to place [2].

Malaria and anemia remain major public health concerns globally and in Ethiopia. Nearly half of the world’s population is at risk of malaria, with 247 million cases and 619,000 deaths reported in 2021 across 85 countries [3]. While malaria is present in World Health Organization (WHO) regions such as Southeast Asia, Eastern Mediterranean, and South America, the African region bears the greatest burden—accounting for 95% of global malaria cases and 96% of deaths [4]. In Ethiopia, malaria continues to be a leading cause of morbidity and mortality, contributing significantly to outpatient visits and hospitalizations [5]. Approximately 60% of the Ethiopian population lives in malaria-risk areas, particularly those located below 2000 meters above sea level [6].

Anemia also poses a significant global health challenge, affecting an estimated 1.62 billion people worldwide [13]. Developing countries account for about 43% of cases, with children comprising 47.4% of the affected population. In Ethiopia, the 2016 Ethiopian Demographic and Health Survey (EDHS) reported that 44% of children are anemic—21% with mild, 20% with moderate, and 3% with severe anemia. The prevalence is higher in rural areas (45%) than in urban areas (35%) [14].

The recurrent occurrence of malaria and anemia were major sources of morbidity and mortality in children particularly in sub-Saharan Africa [7]. Though the predisposing factors of anemia in children were diverse, in tropical countries malaria is a fundamental one that contributes the highest proportion (51.4%) of anemia in young children [8]. The anemia was due to reduced hemoglobin (Hb) concentration since infection with malaria causes hemolysis of both infected and uninfected red blood cells (RBCs) resulting in bone marrow dyserythropoiesis [9].

Anemia occurs primarily due to reductions in the Hb level in the peripheral blood below the normal threshold set for a particular population [10]. Its etiology is multi-factorial including infectious agents such as malaria and Hookworm, severe micronutrient deficiencies, particularly folate, iron and Vit B 12 deficiencies [11]. The consequences, among children—particularly school-aged children is associated with impaired cognitive development, reduced physical growth, weakened immunity, and poor academic performance, making it a major public health concern in this age group. [12].

These days, the malaria elimination program has received the utmost global and national priorities [4]. In tropical areas, malaria control measures have significant impacts on the overall reduction of anemia, and as a result, the malaria control program is considered to be a collateral strategy for the reduction of anemia particularly among highly susceptible groups [15]. Likewise, Ethiopia has given considerable attention to the malaria elimination program intending to maintain the current gains and accelerating the progress toward elimination targets [16]. Hence, malaria elimination program has a great advantage for anemia prevention and control efforts suggesting the need to carefully combine anemia prevention strategies with malaria elimination initiatives. In malaria endemic settings and areas with recently reduced malaria transmission, many children with malaria parasitemia are asymptomatic, thus malaria remains undiagnosed.

## Materials and methods

### Study area

This study was conducted in public primary schools in Hawassa City, the capital of the Sidama National Regional State, located 275 km south of Addis Ababa. The city is divided into 8 administrative sub-cities and further divided into 20 urban and 12 rural kebeles. According to the 2023 report from the Central Statistical Agency (CSA), Hawassa City had a population of 577,075, with 287,734 males and 289,341 females, and 96,720 households [17]. There are 88 healthcare facilities in the city, including 1 public comprehensive specialized hospital, 1 general public hospital, 2 public primary hospitals, 4 private hospitals, 11 public health centers, 17 health posts, and 52 private clinics. Additionally, Hawassa has 48 public primary schools serving 64,226 students, including 27,652 boys and 36,574 girls. Hawassa is a priority city for malaria elimination efforts, and the city’s health department has distributed 74,600 ITNs to households [18].

### Study design and period

A cross-sectional study was conducted from April to June 2024 in selected Hawassa City public primary schools.

### Ethics approval

Ethical approval (Ref No. HU/CMHS/IRB/169/16; dated March 15, 2024) was obtained from the Institutional Review Board (IRB) of Hawassa University College of Medicine and Health Science. Participation was fully voluntary and written informed consent was secured from parents/guardians, and verbal assent was obtained from children. Strict confidentiality was kept during data collection and report writing.

### Sample size and sampling technique

To determine the sample size for this study, the prevalence of malaria and anemia were considered and calculated separately and the largest sample size was taken. Accordingly, the sample size for the prevalence of malaria and anemia was calculated using a single population proportion formula with the assumptions of: prevalence of malaria (11.1%) and anemia (10.9%) [19], 95% confidence level, 5% margin of error and 10% non-response rate as follows:


$$n=\frac{Z_{\frac{a}{2}}^{2}p(1-q)}{d^{2}}$$

Where: n = Minimum sample size required

$Z_{\frac{a}{2}}$ = Standard normal distribution corresponding 95% confidence interval = 1.96,

P = Prevalence of malaria (11.1% = 0.111) and prevalence of anemia (10.9%=0.109),

q = (1 - p),

d = Margin of error = 5%

Accordingly, the sample size for malaria, **n**_**1**_ = 152. Then, by adding 10% non-response rate= (152 + 15.2) = 167.2 ≅ 167

Likewise, the sample size for anemia, **n**_**2**_ = 149. Then, by adding 10% non-response rate= (149 + 14.9) = 163.9 ≅164.

Therefore, the largest sample size of 167 was taken. To account for potential variability due to the multi-stage sampling technique, a design effect of 2 was applied, increasing the final sample size to 334 school children. Since the total population of students across the nine schools included in the study is 25,016, which exceeds 10,000, a population correction formula was not applied.

### Sampling technique

Multi-stage sampling technique was used, starting with stratified sampling where sub-cities were grouped based on their proximity to Lake Hawassa. Then sub-cities were selected randomly within each stratum, ensuring each sub-city had an equal chance of being included. Next, schools from the chosen sub-cities were also selected using lottery method. To ensure a representative sample, proportional allocation was applied for all selected schools. Finally, within each selected school, systematic random sampling was used to select students from the school rosters. The k-value (sampling interval) was determined by dividing the total number of students in each school by the number of students to be selected. Students who meet the inclusion criteria were then chosen at regular intervals (every 75^th^ student) from the roster, stratified by age (6–15 years) and grade levels (1–4 and 5–8) until the achievement of the sample size.

### Data collection

A structured questionnaire involving socio-demographic and risk factors associated with malaria infection and anemia was developed in English and then translated to local languages (Amharic and Sidamu Afoo). Before implementation, the questionnaire was explained to the laboratory technicians responsible for data collection at each school. The principal investigator supervised the data collection process. The purpose of the study was explained to the study participants and their parents/caregivers. This questionnaire was administered to the voluntary study participants’ parents/caregivers during blood sample collection.

### Laboratory investigations

#### Blood film preparation and examination of Malaria Parasites.

Clean slides were labeled at the frosted end with the student’s code. Blood samples were collected from the middle or ring finger after the area was cleaned with a 70% alcohol-moistened swab and dried with cotton. A disposable blood lancet was used to prick the finger, and the first drop of blood was wiped away. Both thick and thin smears were prepared then slides were air-dried at the study site and transported daily to the Alamura Primary Hospital laboratory. The thin blood films were fixed using absolute methanol for 30 seconds before being stained with a 10% Giemsa solution for 10 minutes. After staining, the slides were rinsed with water and air-dried, ready for examination under a microscope. Parasite density was estimated from the thick blood film by counting the number of asexual parasites in relation to white blood cells (WBC), using 200 WBCs unless the parasite count was less than 10 per 200 WBC, in which case 500 WBCs were counted. The WBC count of 8,000/µl was employed to determine parasite density. According to WHO guidelines, parasite load in malaria is categorized into three levels based on the number of parasites per µl of blood. Low: less than 1,000 parasites/µL of blood, moderate: between 1,000 and 10,000 parasites/µL of blood, High: greater than 10,000 parasites/µL of blood [20].

#### Malaria Rapid Diagnostic Test (RDT).

Abbott SD Bioline Malaria Ag (Pf-HRP2 and Pv-pLDH) was used in the field to screen children for possible malaria infection. It is a three-banded device containing a *P. falciparum* HRP2, a *P. vivax* pLDH, and control bands, manufactured by Standard Diagnostics Inc. (now Abbot Diagnostic Korea Inc.) with LOT number: 05DDI013A, and product code 05fk80, Korea. After labeling the device with the student’s code, 5 µL of blood samples were collected for onsite testing. A blood sample was applied to the designated sample well of the test device and two drops of buffer solution were then added to the buffer well to lyse the cells, release antigens, and promote the antigen-antibody reaction. The kit provided results in 15 minutes and interpreted (per the manufacturer’s instructions) [21].

#### Hematological analysis.

Mission® Hemoglobin Meter (with curves product date: 2023-10-05 and expiry date: 2025-10-05; lot number: Hb3100; REF C131-3021) was used to measure the Hb concentration (in g/dL) from finger-pricked blood samples. According to WHO recommendations, anemia is then categorized based on Hb concentration, in children between 5 and 11 years old, and is classified as follows: – Mild: 11–11.4 g/dL, Moderate: of 7–10.9 g/dL, Severe: below 7 g/dL. Likewise, for children aged 12–16 years, mild: 11–11.9 g/dL, moderate: 7–10.9 g/dL, and severe: below 7 g/dl [22].

### Quality assurance

In this study, several measures were taken to ensure data quality. Clinical data and specimen collectors were thoroughly instructed to maintain consistency and accuracy, strictly adhering to standard operating procedures (SOPs) and manufacturers’ guidelines for all diagnostic tests. A pretest was conducted on 5% of the total sample size (17 students) at Edget Fana Primary School. To further guarantee data accuracy, socio-demographic data was double-entered into Epi-info version 7 and verified. Laboratory analyses involved blood slide examinations under oil immersion at 100x magnification by two microscopists, with a third technician resolving any discrepancies. Slides were categorized based on parasite density following WHO guidelines and discrepancies between RDT and microscopy results were rechecked by an additional microscopist. Also, malaria positive, and negative blood films from known malaria-positive and negative blood samples were prepared and used for quality control of the 10% Giemsa stain. Rapid test kits and test strips were checked for expiration dates, correct collection procedures and samples, and in-built control appearances. All results were accurately recorded in a spreadsheet, and regular checks were performed to ensure consistency. Additionally, questionnaires were reviewed daily for completeness, and laboratory test results were properly recorded and managed.

For RDTs, we ensured that all test kits were used within their expiry date and stored according to the manufacturer’s recommendations. Each test was interpreted only if the control band appeared, as per the manufacturer’s instructions. For the hemoglobinometer, although it lacks an internal control system, we ensured regular calibration checks. Additionally, the same operator conducted all measurements to minimize inter-user variability, and results outside expected physiological ranges were rechecked.

### Data analysis

The data for this study was entered into Epi-info version 7, where it was reviewed and cleaned before being transferred to Statistical Package for the Social Sciences (SPSS) version 27 for statistical analysis. Descriptive statistics were used to analyze socio-demographic characteristics and the prevalence of malaria and anemia. Categorical variables were reported as frequencies and percentages, while continuous variables were summarized into means and standard deviations. To assess the risk factors associated with malaria and anemia, binary logistic regression was employed. First, a bivariate logistic regression analysis was performed, and variables with a p-value of ≤ 0.25 were selected for further analysis. These variables were then entered into a multivariable logistic regression model using stepwise selection to control for potential confounders. Variables with p-values < 0.05 were considered statistically significant. The results of the logistic regression were presented as odds ratios with 95% CI.

### Operational definition

#### School children.

Individuals between the ages of 6 to 15 years who are enrolled in school.

#### Malaria-related anemia.

Anemia caused by malaria infection, identified by the presence of malaria parasites and low Hb levels, according to WHO standards.

#### *Plasmodium* parasite density.

The concentration of *Plasmodium* parasites in blood, measured by counting the number of parasites per microliter (µL) of blood using a thick blood film

## Results

### Socio-demographic characteristic

A total of 334 schoolchildren were originally enrolled in the study; however, five participants were excluded based on specific exclusion criteria, resulting in a response rate of 98.5%. Consequently, 329 children were included in the final analysis. Among these participants, 57.4% (189) were female, with a mean age of 11.93 years (±2.4 SD). Over half of the children, 55.3% were in grades (5–8), while 44.7% were in grades (1–4). Additionally, 60.2% (198) attended schools located in sub-cities close to the lake, while 39.8% (131) in sub-cities situated distant from it (Table 1).

**Table 1:** Socio-demographic characteristics of primary school children in Hawassa City, Sidama, Ethiopia, 2024.

Characteristics of participants	Category	n (%)
Students Sex	Male	140 (42.6)
	Female	189 (57.4)
Students Age	6-11	134 (40.7)
	12-15	195 (59.3)
Grade Level	1-4	147 (44.7)
	5-8	182 (55.3)
Occupation of caregiver	Merchant	85 (25.8)
	Civil servant	90 (27.4)
	Daily laborer	59 (17.9)
	Farmer	17 (5.2)
	House wife	34 (10.3)
	Others^*^	44 (13.4)
Educational status of caregiver	Unable to read and write	11 (3.3)
	Primary school	154 (46.8)
	High school	117 (35.6)
	Higher Education	47 (14.3)
Monthly income of caregiver (ETB^**^)	<1000	5 (1.5)
	1000-2000	89 (27.1)
	2001-3000	80 (24.3)
	3001-4000	88 (26.7)
	> 4000	67 (20.4)

* Others: -private business owners, drivers, housekeepers (not full-time), and unemployed individuals.

** ETB:- Ethiopian Birr.

### Prevalence of Malaria

The overall prevalence of malaria among the 329 school children from 9 public primary schools was 8.5% (95% CI: (5.5% − 11.5%). Microscopy revealed a malaria prevalence of 4.86% (95% CI: 2.6%− 7.4%). As shown in Table 2, most malaria-infected students, 3.34% (11/16), were in the (12–15) age groups. Malaria prevalence among males was 1.82% (6/16), with 3.34% (11/16) of cases occurring in schools near the lake. In grades (5–8), 3.03% (10/16) of malaria cases were reported. *P. falciparum* accounted for 56.25% of infections, while *P. vivax* made up 43.75%. Among *P. falciparum*-infected students, 44.4% were males and 55.6% were females.

**Table 2:** Prevalence of malaria, anemia, and parasite density by age, sex, and grade level among primary School children in Hawassa City, Sidama, Ethiopia, 2024.

	RDT n (%)		Microscopy n (%)		Parasite density n (%)			Anemia status n (%)	
	***P.f***	***P.v***	***P.f***	***P.v***	**<1000**	**1000−10,000**	**>10,0000**	**Anemic**	**Non-anemic**
Sex									
Male	7(2.1)	3(1.0)	4(1.2)	2(0.6)	2(0.6)	4(1.2)	0	14(10)	126(90)
Female	8(2.4)	4(1.2)	5(1.5)	5(1.5)	8(2.4)	2(0.6)	0	17(9)	172(91)
Age									
6-11	5(1.5)	3(1.0)	3(1.0)	2(0.6)	3(1.0)	2(0.6)	0	11(8.2)	123(91.8)
12-15	10(3.0)	4(1.2)	6(1.8)	5(1.5)	7(2.1)	4(1.2)	0	20(10.3)	175(89.7)
Grade level									
1-4	6(1.8)	3(1.0)	3(1.0)	3(1.0)	5(1.5)	1(0.3)	0	15(4.6)	132(40.1)
5-8	9(2.7)	4(1.2)	6(1.8)	4(1.2)	5(1.5)	5(1.5)	0	16(4.9)	166(50.4)
Total	15(4.6)	7(2.1)	9(2.7)	7(2.1)	10(3.0)	6(1.8)	0	31(9.4)	298(90.6)

*P.f- Plasmodium falciparum P.v- Plasmodium vivax.*

RDT revealed a malaria prevalence of 6.7% (95% CI: 4.0% to 9.4%). Among the children diagnosed, 15 (4.6%) were infected with *P. falciparum* and 7 (2.1%) with *P. vivax*, while 307 (93.3%) tested negative. The infection rate was almost equal between genders, with females making up 12 (3.7%) of the cases. Children aged 6–11 years accounted for 8 (2.4%) of the infections. A higher prevalence was noted in students attending schools near the lake, with 17 (5.2%) of the cases. Malaria prevalence across schools ranged from 0.0% to 20% (Table 3).

**Table 3:** Participation and malaria positivity among primary School children in Hawassa City, Sidama, Ethiopia, 2024.

		*Plasmodium* species +ve by microscopy		*Plasmodium* species +ve by RDT	
Name of school	No. students selected	*P. falciparum*	*P. vivax*	*P. falciparum*	*P. vivax*
Argo Hameso	45(13.7%)	2	1	4	1
Diaspora	20(6.1%)	0	0	0	0
Ethiopia Tikdem	28(8.5%)	1	1	0	1
Gebeya Dar	10(3.0%)	2	0	3	0
Gimb Genet	32(9.7%)	0	1	0	2
Gudumale	58(17.6%)	0	2	2	1
Hogowa	57(17.3%)	2	1	3	2
Nigist Fura	54(16.4%)	1	1	2	0
Stadium	25(7.6%)	1	0	0	1
Total	329(100%)	9	7	15	7

When comparing the two diagnostic methods, RDT identified a slightly higher malaria prevalence of 6.7%, detecting more *P. falciparum* cases (15) than microscopy (9). Students from schools near Lake Hawassa had higher infection rates; a trend confirmed by both RDT and microscopy results. Additionally, older children (12–15 years) had a higher prevalence of malaria compared to younger ones (6–11 years), and females had a slightly higher infection rate than males, as observed with both diagnostic methods (Table 3).

*Plasmodium* asexual parasite densities were determined for all slide positive children, and ranged from 80 to 2,040 parasites/µL. Low-density *Plasmodium* infections were found in 62.5% (10/16) cases, of which 50% (5/10) were anemic. Furthermore, it was observed that more than half of these children were gametocyte carriers, the infective stage of *Plasmodium* species to female *Anopheles* mosquitoes.

### Hemoglobin measurement

**Fig 1:**
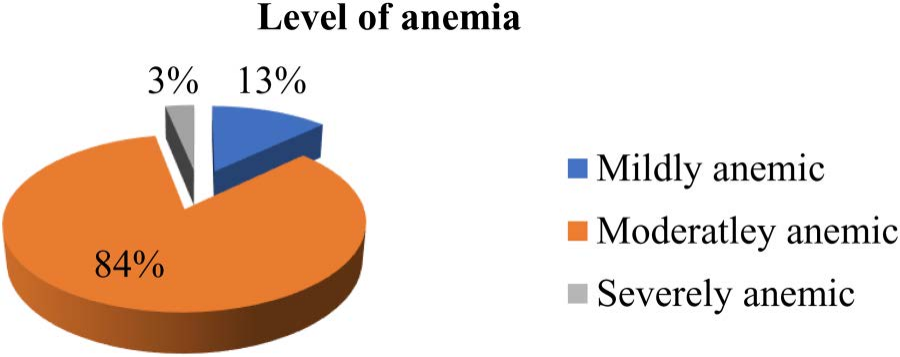
Level of anemia in percentage among anemic primary School children in Hawassa city, Sidama, Ethiopia, 2024.

Hemoglobin concentrations, used to assess anemia, ranged from 6.9 to 16.6 g/dl, with a mean of 13.7 g/dl (± 1.81 SD). The overall prevalence of anemia among the school children was 9.4% (95% CI: 6.2% to 12.6%). Of the anemic children, 4 (12.9%) were mildly anemic, 26 (83.9%) were moderately anemic, and 1 (3.2%) was severely anemic. Fig 1 illustrates the distribution of mild, moderate, and severe anemia among the affected children. Anemia was more prevalent in females, representing 54.8% of cases, and it was most common (64.5%) in the 12–15 age group. In children from grades 1–4, anemia accounted for 48.4% of cases.

**Fig 2:**
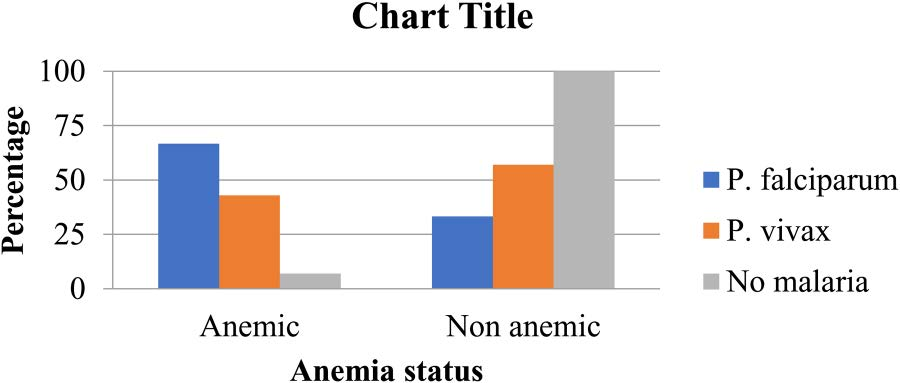
Distribution of *Plasmodium* species among primary School children with different anemia status in Hawassa city, Sidama, Ethiopia, 2024.

Additionally, 29% (9/31) of the anemic children were diagnosed with malaria through microscopic examination. Within this malaria-infected group, 11.1% (1/9) had mild anemia, 77.8% (7/9) had moderate anemia, and 11.1% (1/9) had severe anemia. Of the malaria-infected children, 66.7% (6/9) had *P. falciparum*, and 33.3% (3/9) had *P. vivax* (Fig 2). On the other hand, malaria diagnosis through RDT revealed that 35.5% (11/31) of the anemic children had malaria. Within this group, 90.9% (10/11) had moderate anemia, and 9.1% (1/11) had severe anemia. Among the malaria-infected children, 72.7% (8/11) were infected with *P. falciparum*, while 27.3% (3/11) were infected with *P. vivax*. Conversely, 90.6% (298/329) of the children had normal Hb levels according to WHO classification.

### Factors associated with malaria infection in school children

Initially, bivariate analysis factors such as caregiver education and income, ITN use, stagnant water near the household, the child’s history of malaria or anemia, and caregiver knowledge of malaria transmission showed significant links to malaria infection.

As shown in Table 4, multivariate logistic regression analysis revealed five factors significantly associated with malaria (P < 0.05). Anemic children had nearly 5 times the odds of being infected with malaria compared to non-anemic children [AOR = 4.983, 95% CI: 1.067–23.265]. Similarly, children with a previous history of malaria were 9 times more likely to contract the disease [AOR = 9.121, 95% CI: 1.686–49.336]. Children without a family history of malaria had a 90% higher likelihood of infection compared to those from families with a history of the disease [AOR = 0.098, 95% CI: 0.015–0.633]. Caregivers who have knowledge of how malaria is transmitted are about 80% less likely to be infected compared to those who lack this knowledge [AOR = 0.205, 95% CI: 0.049–0.854]. Furthermore, the presence of ITNs in households significantly lowered the risk of malaria, with children from homes using ITNs being 98% less likely to be infected [AOR = 0.024, 95% CI: 0.001–0.755].

**Table 4:** Bivariate and multivariate analysis of factors associated with malaria among primary School children in Hawassa city, Sidama, Ethiopia, 2024.

Individual child factors Categories	All	n (%)		COR (95% CI)	p-value	AOR (95% CI)	p-value
		+ve	−ve				
**Age**							
6-11	134	5	129	1.542 (0.523,4.546)	0.432		
12-15	195	11	184	1			
**Sex**							
Male	140	6	134	1.248 (0.443,3.518)	0.676		
Female	189	10	179	1			
**Grade level**							
1-4	147	6	141	1.366 (0.485,3.851)	0.555		
5-8	182	10	172	1			
**Educational status of caregiver**							
Can`t read and write	11	3	8	6.000 (1.355,26.570)	0.018		
1-8	153	9	144	14.250 (2.468,82.293)	0.003		
9-12	117	3	114	17.625 (1.625,191.216)	0.018		
Tertiary	48	1	47	1			
**Income of caregiver**							
<1000	5	3	2	0.010 (0.001,0.145)	0.001		
1000-1500	89	8	82	0.155 (0.019,1.273)	0.083		
2000-2500	80	3	76	0.384 (0.039,3.779)	0.412		
3000-3500	88	1	87	1.318 (0.081,21.466)	0.846		
> 4000	67	1	66	1			
**ITN usage**							
Yes	108	1	107	0.128 (0.017,0.985)	0.048	0.024 (0.001,0.755)	0.034
No	221	15	206	1		1	
**Stagnant water surrounding household**							
Yes	119	9	110	2.373 (0.860,6.545)	0.095		
No	210	7	203	1			
**Child diagnosed with malaria before**							
Yes	110	12	98	6.582 (2.070,20.922)	0.001	9.121 (1.686,49.336)	0.010
No	219	4	215	1		1	
**Child diagnosed with anemia before**							
Yes	98	7	91	1.897 (0.686,5.248)	0.217		
No	231	9	222	1			
**Caregivers’ knowledge on how malaria transmitted**							
Yes	204	5	199	0.260 (0.088,0.768)	0.015	0.205 (0.049,0.854)	0.030
No	125	11	114	1		1	
**Household history of malaria**							
Yes	174	3	171	0.192 (0.054,0.686)	0.011	0.098 (0.015,0.633)	0.015
No	155	13	142	1		1	
**Anemia status**							
Yes	31	9	22	17.006 (5.783,50.010)	0.001	4.983 (1.067,23.265)	0.041
No	298	7	291	1		1	
**Clean drinking water access**							
Yes	272	10	262	0.324 (0.113,0.932)	0.037		
No	57	6	51	1			

### Factors associated with anemia in schoolchildren

In the bivariate analysis, several factors were found to be associated with anemia among school age children. These included the caregiver’s education and income levels, access to clean drinking water, and the child’s history of malaria or anemia. Additionally, the caregiver’s knowledge of anemia causes, their nutritional knowledge, and the child’s history of wasting were significant contributors. Other factors related to anemia included whether the child received iron or vitamin supplements, meal frequency, the habit of skipping meals, and the presence of malaria infection.

To identify independent predictors of anemia, all explanatory variables with a P-value ≤ 0.25 in the bivariate analysis were entered into a multivariate logistic regression model. The multivariate analysis revealed that children who ate two meals per day had 6.24 times the odds of being anemic compared to those with more frequent meals [AOR = 6.243, 95% CI: 1.956–19.923]. Similarly, the odds of anemia were 5.8 times higher among children with a history of wasting compared to those without [AOR = 5.760, 95% CI: 2.059–16.112]. Malaria-infected school children were over 13 times more likely to be anemic than non-infected [AOR = 13.258, 95% CI: 3.188–55.139] (Table 5).

**Table 5:** Bivariate and multivariate analysis of factors associated with anemia among primary school children in Hawassa city, Sidama, Ethiopia, 2024.

Individual child factors Categories	All	n (%)		COR (95% CI)	p-value	AOR (95% CI)	p-value
**+ve**	**−ve**		
**Age**							
6-11	134	11	123	1.278 (0.591,2.763)	0.533		
12-15	195	20	175	1			
**Sex**							
Male	140	14	126	1.124 (0.534,2.365)	0.758		
Female	189	17	172	1			
**Grade level**							
1-4	147	15	132	1.179 (0.562,2.472)	0.663		
5-8	182	16	166	1			
**Educational status of caregiver**							
Can`t read and write	11	4	7	0.076 (0.012,0.496)	0.007		
1-8	153	22	131	0.259 (0.059,1.144)	0.075		
9-12	117	3	114	1.652 (0.267,10.214)	0.589		
Tertiary	48	2	46	1			
**Income of caregiver**							
<1000	5	3	2	0.031 (0.004,0.263)	0.001		
1000-2000	89	17	73	0.201 (0.056,0.719)	0.014		
2001-3000	80	5	74	0.694 (0.160,3.017)	0.626		
3001-4000	88	3	85	1.328 (0.259,6.798)	0.733		
> 4000	67	3	64	1			
**Clean drinking water access**							
Yes	272	22	250	0.469 (0.204,1.081)	0.076		
No	57	9	48	1			
**Child diagnosed with malaria before**							
Yes	110	17	93	2.677 (1.266,5.659)	0.010		
No	219	14	205				
**Child diagnosed with anemia before**							
Yes	98	14	84	2.098 (0.990,4.446)	0.053		
No	231	17	214				
**Knowledge on how anemia caused**							
Yes	106	7	99	0.586 (0.244,1.407)	0.232		
No	223	24	199	1			
**Nutritional knowledge of caregiver**							
Yes	113	7	106	0.528 (0.220,1.267)	0.153		
No	216	24	192	1			
**Wasting history**							
Yes	87	18	69	4.595 (2.144,9.850)	0.001	5.760 (2.059,16.112)	0.001
No	242	13	229	1		1	
**Iron and vitamin supplement for the child**							
Yes	33	1	32	0.277 (0.037,2.101)	0.214		
No	296	30	266				
**Meal frequency per day**							
Two times	28	10	18	7.639 (3.117,18.722)	0.001	6.243 (1.956,19.923)	0.002
Three times	295	20	275	2.778 (0.284,27.211)	0.380	4.779 (0.257,89.020)	0.295
Four times & above	6	1	5	1		1	
**Meal skipping habit**							
Yes	109	14	95	1.760 (0.833,3.718)	0.139		
No	220	17	203	1			
**Malaria infection**							
Yes	16	9	7	17.006 (5.783,50.01)	0.001	13.258 (3.188,55.139)	0.001
No	313	22	291			1	

## Discussion

In this study, the prevalence of malaria was found to be 4.86%, as determined through microscopy. The findings regarding malaria prevalence in this study align with those from a previous study among school-aged children conducted in Southern Ethiopia, which reported a prevalence of 3.6% [23], and higher from a study conducted in Gamo and Gofa zone, (1.62%) [2]. In contrast the prevalence of malaria in the present study area was lower than findings in Gedeo (11.1%) [19],Tanzania (18.4%) [24], Cameroon (41.7%) [25] and Ghana (59%) [26]. The differences in malaria prevalence among studies might be attributed to environmental and seasonal variations. For example, our study was conducted during the minor transmission season (April to June), which likely contributed to the relatively lower malaria prevalence compared to studies conducted during peak transmission seasons in regions such as Gedeo and Tanzania. Additionally, the ecological setting of our study area—Hawassa City—includes schools near Lake Hawassa, where mosquito breeding conditions may differ from those in highland or arid regions.

Among malaria positive children in this study, the prevalence of *P. falciparum* was 56.25%; this is almost similar to a study conducted in East Shewa Zone 57.1% [27] but lower than Ghana 97% [26] and Jimma 90% [6]. However, it is high compared to a study in Dilla 45.7% [19]. On the other hand, the prevalence of *P. vivax* in this study was 43.75%, which is comparable to the 41% prevalence reported in East Shewa Zone [27]. Regarding parasitemia levels, the majority of parasitemic children had low parasite densities. This may indicate that these children have developed some degree of immunity through previous malaria exposure, leading to less severe infections with lower parasitemia levels.

Although the difference between RDT- and microscopy-detected malaria cases appears small in proportion, it is important to consider that many of the cases detected by RDT but not by microscopy likely had low parasitemia levels, which can be missed by microscopy due to its lower sensitivity in such cases. Moreover, RDTs based on HRP2 antigen detection can remain positive for days to weeks after parasite clearance, further explaining the discrepancy. Therefore, the observed difference is not solely due to test accuracy but also to biological and diagnostic factors inherent to each method.. Both methods confirmed higher infection rates in students near Lake Hawassa, likely due to the favorable conditions for mosquito breeding around the lake. Older children (12–15 years) exhibited a higher prevalence than younger ones (6–11 years), which may be linked to increased outdoor activities. Females also showed slightly higher infection rates than males, potentially reflecting differences in exposure, and protective practices.

Anemic school children were at a significantly higher risk of having asymptomatic malaria infection, consistent with findings from previous studies [6,24,33]. A possible explanation is that anemia weakens the immune system, making it harder for the body to fight malaria parasites, thus leaving the children more susceptible to serious infections. Similarly, an association was observed between malaria and caregivers’ knowledge of how it is transmitted. This could be a lack of knowledge may lead to higher transmission rates, as caregivers might not take the necessary steps to protect children from mosquito bites or could delay seeking treatment when symptoms appear. Another result of this study is that children who had a family history of malaria were 90% less likely to be infected with malaria infection than those with no history. This is mainly because children who have a family history of malaria are more likely to have been exposed to the disease in the past; this exposure helps them build up their acquired immunity and makes them less likely to contract the disease [6]. Also, children with a prior malaria diagnosis exhibit significantly higher odds of malaria. This might be due to repeated exposure to the malaria parasite, which heightens the likelihood of reinfection. School children living in households without ITNs were more likely to contract malaria compared to those in households with ITNs, which is consistent with previous studies [27,28]

One of the consequences of malaria is anemia since the parasites destroy RBC after completing the developmental life cycle [25]. Malaria is associated with anemia and its insidious nature of presentation means that mild to moderate degrees of anemia frequently remain undetected and untreated [2]. Studies have shown that long-term asymptomatic malaria could lead to anemia. This study also, demonstrates a significant association between malaria and anemia.

The mean Hb concentration among school children was 13.7 g/dL, with a corresponding anemia prevalence of 9.4%. The findings from this study showed a lower prevalence of anemia compared to similar population groups in the Gamo and Gofa zones (37.3%) [2], Tanzania (19.8%) [24], and Cameroon (56.2%) [25]. This difference may be due to several factors, including variations in nutritional status, healthcare access, and the prevalence of infectious diseases in different regions. Areas with higher levels of poverty and food insecurity are often associated with greater rates of anemia due to insufficient dietary iron and other essential nutrients. Conversely, the prevalence of anemia in this study was higher than the estimate made among school-age children in Lao People ’ s Democratic Republic (6.3%) [29], and Jimma (5.8%) [6]. The lower prevalence in these regions may suggest more effective public health interventions, better nutritional programs, or less exposure to malaria and other infectious diseases that contribute to anemia. Consistent with our findings study done in Dilla resulted in (10.9%) [19]. This alignment may indicate comparable levels of nutritional status, exposure to malaria, and healthcare access among school children in both areas.

This study found that children with a wasting history are more likely to be anemic than children without a wasting history and children’s meal frequency per day is a determinant factor for anemia This might be due to wasting leads to malnutrition, which impairs RBC production. Additionally, meal frequency is a key factor for anemia, as regular, nutritious meals provide essential nutrients needed to prevent anemia Literature indicates there is indeed a strong association of malaria with increased prevalence of anemia through several mechanisms including the destruction of RBC, our study finds an association between anemia and malaria infection. This finding is supported by the study done in Benishangul-Gumuz Region [30], Tanzania [24], Cameroon [25], Nigeria [1] which showed that anemia was significantly associated with malaria. The possible explanation for this could be that malaria is one of the major causes of anemia by causing increased destruction of both parasitized and un-parasitized RBC (immune-mediated hemolysis, phagocytosis, splenic sequestration) and by decreasing production of RBCs in the bone marrow (bone marrow suppression, inadequate reticulocyte production, effects of inflammatory cytokines and effects of parasite factors) [9].

The prevalence of malaria and anemia was not associated with age, sex, or grade level. This finding is consistent with a study conducted in Cameroon, which found that the prevalence of anemia during asymptomatic malaria parasitemia was not associated with age, sex, or socioeconomic status [25,31]. In contrast, other studies found that age and sex were related to the prevalence of asymptomatic malaria in eastern Ethiopia [32].

## Conclusion

This study revealed that both malaria and anemia remain important public health problems among school children in Hawassa City, with a prevalence of 8.5% and 9.4%, respectively. The co-existence of these conditions, even in an urban setting, underscores the ongoing vulnerability of school-aged children. Significant associations were found between malaria or anemia and factors such as residence, ITN usage, and nutritional status. These findings call for integrated and targeted interventions that address both malaria prevention and nutritional health, particularly in peri-urban areas. School-based screening and health education programs should be strengthened to reduce the burden and improve children’s overall well-being.

## Supporting information

S1 Questionnaire. (DOCX)
