# Supplementary material for: Prevalence of malaria, anemia and associated factors among school children in Hawassa city, Sidama, Ethiopia
Source: PLoS One. 2025 Jul 17;20(7):e0327378. doi: 10.1371/journal.pone.0327378 (PMC12270185; doi:10.1371/journal.pone.0327378)
Supplement: S1 — (DOCX) [file pone.0327378.s001.docx]

**Questionnaire**

Questionnaire for the demographic characteristics and investigation of the prevalence of malaria and anemia and associated factors among school children in Hawassa City, Sidama, Ethiopia.

| **Part one: Personal Information** | | |
| --- | --- | --- |
| **S. no** | **Items** | **Response** |
| Q101 | Sex | Male |
|  |  | Female |
| Q102 | Age in years |  |
| Q103 | Grade level | 1-4 |
|  |  | 5-8 |
| **Part two: Socioeconomic Status** | | |
| Q201 | Occupation of mother/primary caregiver? | Merchant |
|  |  | Civil servant |
|  |  | Daily laborer |
|  |  | Farmer |
|  |  | House wife |
|  |  | Other |
| Q202 | Education level of mother/primary caregiver? | Unable to read and write |
|  |  | Primary school |
|  |  | High school |
|  |  | Higher Education |
| Q203 | Monthly income of mother/primary caregiver? | <1000 |
|  |  | 1000-1500 |
|  |  | 2000-2500 |
|  |  | 3000-3500 |
|  |  | > 4000 |
| **Part three: Household Information** | | |
| Q301 | How many people live in your household? | < 4 |
|  |  | ≥4 |
| Q302 | Do you use ITNs (insecticide-treated nets) to prevent mosquito bites during sleep? | Yes |
|  |  | No |
| Q303 | If yes, for Q302 who uses the available ITN(s)? | Children only |
|  |  | Mother only |
|  |  | Mother and Father only |
|  |  | Mother and children only |
|  |  | Whole family |
| Q304 | Do you have access to clean drinking water at home? | Yes |
|  |  | No |
| Q305 | Is there stagnant water surrounding your house? | Yes |
|  |  | No |
| **Part four: Health History** | | |
| Q401 | Has the child ever been diagnosed with malaria? | Yes |
|  |  | No |
| Q402 | Has the child ever been diagnosed with anemia? | Yes |
|  |  | No |
| Q403 | Has the child received any treatment for malaria in the past two weeks? | Yes |
|  |  | No |
| Q404 | Has the child been diagnosed with a worm infection in the past month? | Yes |
|  |  | No |
| Q405 | Has the child been diagnosed with or shown signs of wasting (thinness) in the past year? | Yes |
|  |  | No |
| Q406 | Does the child frequently experience fatigue or weakness (at least once a week)? | Yes |
|  |  | No |
| Q407 | Does the child have any chronic health conditions (e.g., diabetes, asthma)? | Yes |
|  |  | No |
| Q408 | Does your household have a history of malaria? | Yes |
|  |  | No |
| **Part five: Knowledge and Practices** | | |
| Q501 | Do you know how malaria is transmitted? | Yes |
|  |  | No |
| Q502 | Do you know how anemia is caused? | Yes |
|  |  | No |
| Q503 | Do you have knowledge about proper nutrition and its impact on health? | Yes |
|  |  | No |
| Q504 | Do you practice dietary diversity during food preparation? | Yes |
|  |  | No |
| Q505 | If yes to Q504, which types of products are included in your dietary diversity? | Animal product |
|  |  | Plant product |
|  |  | Both |
| Q506 | Do you give any iron supplements or vitamins regularly to the child? | Yes |
|  |  | No |
| Q507 | Meal frequency of the child per day? | One times |
|  |  | Two times |
|  |  | Three times |
|  |  | Four times and above |
| Q508 | Does the child have a habit of skipping meals? | Yes |
|  |  | No |

.
